# Observation of viscous liquid flow in tobacco substrate during heating using optical coherence tomography

**DOI:** 10.1098/rsos.230150

**Published:** 2023-08-23

**Authors:** Tiara N. Pratiwi, Toshiaki Iwai, Iori Nakaya, I. Wuled Lenggoro

**Affiliations:** ^1^ Department of Food and Energy Systems Science, Tokyo University of Agriculture and Technology (TUAT), Koganei, Tokyo, Japan, Graduate School of BASE, TUAT, Koganei, Tokyo, Japan; ^2^ Department of Biomedical Engineering, Tokyo University of Agriculture and Technology (TUAT), Koganei, Tokyo, Japan, Graduate School of BASE, TUAT, Koganei, Tokyo, Japan; ^3^ Graduate School of BASE, Tokyo University of Agriculture and Technology (TUAT), Koganei, Tokyo, Japan, Graduate School of BASE, TUAT, Koganei, Tokyo, Japan; ^4^ Department of Applied Physics and Chemical Engineering, Graduate School of Bio-Applications and Systems Engineering (BASE), Tokyo University of Agriculture and Technology (TUAT), Koganei, Tokyo, Japan, Graduate School of BASE, TUAT, Koganei, Tokyo, Japan

**Keywords:** fluid, transport, capillary, evaporation, porous

## Abstract

The present study used optical coherence tomography (OCT) to monitor the dynamics of a highly viscous liquid in a porous tobacco substrate during heating. The OCT technique was integrated with a specially designed heating chamber and an air pump for measuring. Two transitional points in the liquid behaviours at different temperatures were estimated using OCT and statistical analysis of the attenuation coefficient. The first point, ‘A’, shows the time approximation at which the penetration-dominant zone transitions into the evaporation-dominant zone. The second point, ‘B’, indicates the time approximation at which rapid evaporation of free liquid transitions into slow evaporation of trapped and bound liquid. This analytical system is an alternative for tracking liquid transport in porous biomass during heating.

## 1. Introduction

Visualizing liquid transport in multilayered porous media is important in applications such as microfluidic analytical devices [1], food packaging [2] and inkjet printing [3]. Visualizing *in-plane* liquid spreading is common [4–6], but visualization of *trans-plane* liquid penetration is challenging due to factors such as the complex geometry of the multilayered porous structure and liquid volatility [7].

To help visualize liquid transmission in multilayered planes, previous studies have proposed methods such as X-ray-computed tomography [8], micro-CT [9], nuclear magnetic resonance (NMR) [10], digital camera-based light reflection measurement [11], light transmission and impedance spectroscopy [3], a micro-model (a representation of a porous medium) combined with confocal laser scan microscopy [12], and optical coherence tomography (OCT) [13–16]. X-ray and micro-CT offer non-destructive visualization of liquid flow in porous media, such as smart textiles [8] and powder beds [9], with a spatial resolution of around 5–150 µm [17]. However, repeated observations may cause potential health hazards due to exposure to X-ray radiation. NMR is another example of a non-invasive imaging technique. Nonetheless, the quality of the obtained data is heavily dependent on the intricate sample preparation, which can limit the practicality of this approach. Digital camera-based imaging technique offers practicality and a wide range of applicability. Nevertheless, it mainly relies on a two-dimensional surface imaging system which may not accurately capture the behaviour of liquid flow in a three-dimensional system. Micro-models are often adapted to study fluid flow and transport phenomena at the pore-scale level of an opaque porous media [12]. The transparent nature of a micro-model allows a straightforward observation of liquid flow in the pore channel. However, the simplification of a pore network by the fabricated pore models raises an issue with the accuracy of its modelling in comparison with a complex natural system.

OCT captures cross-sectional images of materials by emitting a beam of light into the sample and measuring the intensity of the light that is scattered back to the detector. OCT can reach a penetration depth of up to 1 mm with a resolution of up to 1 µm [18]. In previous studies, OCT has been used to monitor viscous liquid sorption into paper [13], drying of colloid droplets [15,16] and evaporation of water from porous tobacco layers/substrates [14]. To the best of our knowledge, all studies on liquid flow using OCT technique were conducted under ambient conditions. This limits the system's ability to study dynamic processes, such as the effect of changing temperature, on the liquid flow in porous media.

Cigarette smoking has been linked to various diseases and disorders [19,20]. In conventional cigarettes, tobacco is burnt at a temperature of 600–900°C. It produces smoke that contains more than 6000 compounds [19]. A new concept of heated tobacco products (HTP)—inspired by the evaporation of nicotine in tobacco leaves at temperatures below 300°C (far below the combustion temperature of conventional cigarettes)—has attracted attention in recent years [21,22]. Viscous liquid such as glycerol is used as humectants and as the main aerosol former, i.e. without the presence of glycerol, the gas–vapour mixtures cannot condense as inhalable aerosol droplets [23,24]. A detailed study on the transport of glycerol in tobacco sheets during heating is yet to be conducted [3,13,25]. This knowledge is needed to understand the control of aerosol formation from HTP so that the system (i.e. heated tobacco device) can be designed to have little impact on indoor air quality. However, it should be noted that lower indoor emissions of HTP compared with conventional cigarettes may not necessarily correlate to lower health risks [22].

Heated tobacco product is an example of applications that necessitate a visualization and analytical system for tracking liquid flow in porous media that can be integrated with a heating system. Other potential applications for such systems may be found in the elucidation of various phenomena in the drying processes of biomass [26].

The present study aims to demonstrate, for the first time, the use of OCT, integrated with a specially designed heating chamber and an air pump, to monitor the behaviour of viscous liquids (e.g. glycerol) in a porous tobacco substrate during heating. The OCT method is flexible, can be integrated with other systems, and can provide tomographic images of samples under atmospheric pressure.

## 2. Experimental set-up

**Figure 1.**
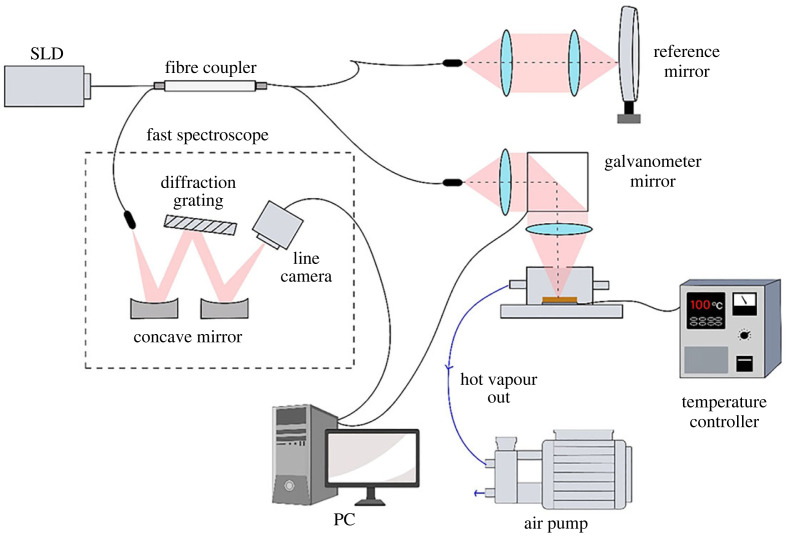
A spectral-domain optical coherence tomography (SD-OCT) system coupled with a heating chamber (with a biomass sample) and an air pump. SLD is a near-infrared superluminescent diode.

The set-up for real-time observation of liquid transport in porous biomass under heating is shown in figure 1. It consists of a spectral-domain optical coherence tomography (SD-OCT) system coupled with a specially designed heating chamber connected to an air pump's suction line. The light source in the OCT system is a near-infrared superluminescent diode (SLD) with a wavelength of 1470 nm and a bandwidth of 44 nm. The depth resolution of the OCT tomogram is 22 µm. The tomogram was obtained by scanning the beam focused on the sample's surface with a galvanometer mirror. The horizontal scanning range was 500 µm with a spot size (diameter of a focused beam of light on the sample surface) of 5 µm. The OCT system scanned the sample in 10 s, from start to finish, for each run.

A reconstituted tobacco sheet (JT, Japan) was used because the reconstitution process yielded a chemically homogeneous sample. The tobacco substrates—a densely connected fibre with a heterogeneous inter-fibre pore size of several micrometres—218 ± 3.5 µm thick and 2.5 × 2.5 cm large. It was fixed on top of the ceramic heater (Sakaguchi E.H Voc Corp., Japan) with heat-resistant tape. The heating temperature was controlled by a desktop-type temperature control unit (Shimaden Co., Ltd, Japan) and was set to heat to 100, 120 and 200°C. The ceramic heater and the sample were mounted inside the heating chamber, which was then placed under the objective lens of the OCT system, as illustrated in figure 1. The temperature profiles of the top and bottom surfaces of the tobacco substrates during the experiment were measured using a T-type ultrafine (*φ* = 0.127 mm) thermocouple (As One Corp., Japan) and recorded in a data logger (Graphtec Corp., Japan).

**Figure 2.**
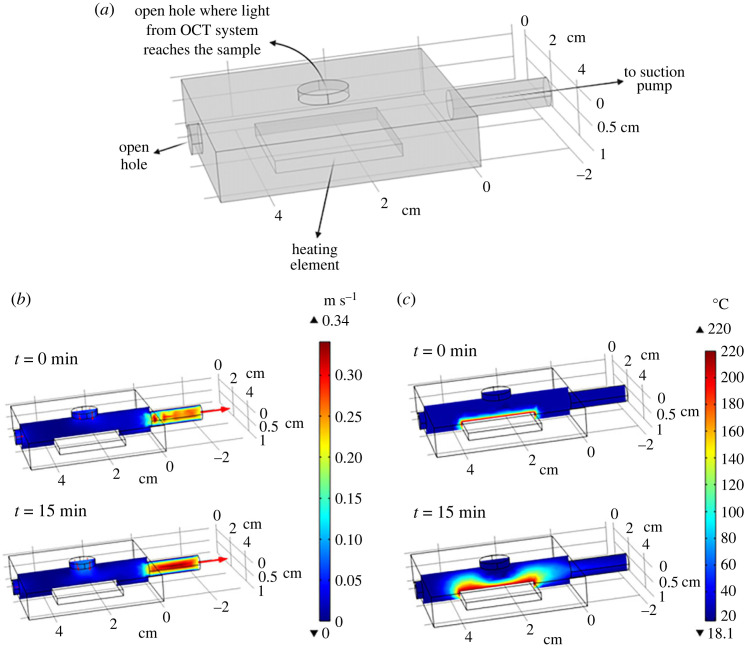
(*a*) Geometry of the heating chamber, for heater temperature set at 200°C and suction velocity of 0.2 m s^−1^, (*b*) calculated velocity profile inside the chamber and (*c*) calculated temperature profile in the chamber after/before operating the suction-pump.

The time-dependent temperature and airflow profile in the chamber during the experiment were simulated using a heat/fluid dynamics analysis software (COMSOL Multiphysics). Figure 2*a* shows the chamber's geometry and dimensions. For the numerical simulation, the heating element was set at 200°C. The hot vapour inside the chamber was pumped at 0.2 m s^−1^ from the right side of the chamber. One side of the chamber and the hole at the top of the chamber were set as open boundaries. The simulation proved that even at temperatures as high as 200°C, suction flow as low as 0.2 m s^−1^ prevented the runaway hot vapour from flowing upward toward the objective lens (figure 2*b,c*). In this experiment, the suction flow rate, set at 6 l min^−1^, caused no condensation on the glass plate temporarily placed above the upper hole of the chamber. This set-up was essential to prevent hot vapour flow to the optical lens, which could affect the results and damage the lens.

At the beginning of each observation, the dry sample was scanned before dropping 20 µl of glycerol (99.5% purity and 290°C boiling point at 1 atm) (Wako Pure Chemicals Industries Ltd, Japan). Heating started simultaneously with the dropping of glycerol on the sample; the observation was completed in 15 min.

The output from the OCT analysis system is backscattered light intensity (*I*_(*z*)_) as a function of depth (*z*) and horizontal scanning length/transverse length. The raw data from the OCT system were processed in MATLAB to construct the matrix for producing the tomographic image and to calculate the attenuation coefficient (*µ_t_*) based on Lambert–Beer's Law [27].

2.1 $$I_{\left(z\right)}=\;I_{0}\exp[-2\;(\mu_{s}^{\prime }+\mu_{a})\;z]$$

and

2.2 $$-\frac{1}{2}\frac{d}{dz}\log\frac{I_{\left(z\right)}}{I_{0}}=\;\mu_{s}^{\prime }+\mu_{a}=\mu_{t},$$

where *I*_0_ is the incident light intensity, *z* is the optical path length, *µ_a_* is the absorption coefficient, and $\mu_{s}^{\prime }$ is the equivalent scattering coefficient. The constructed matrix for the tomographic image was then plotted in OriginPro (OriginLab) to produce the final contour of the tomogram.

Duncan's multiple range test was conducted to estimate the transitional points for the transport (penetration and evaporation) of glycerol in porous biomass using the International Business Machines Corporation's Statistical Package for the Social Sciences (IBM SPSS) software. We coated the sample with platinum thin films using the sputtering process (Smart Coater, JEOL Ltd, Japan) and observed the morphology before and after heating with a scanning electron microscope (SEM JSM 6510, JEOL Ltd, Japan).

## 3. Results and discussion

Barontini *et al*. [28,29] noted that coupling thermogravimetric analysis and Fourier transform infrared spectroscopy (FTIR) gas analysis of tobacco at low heating rates resulted in four regions of weight loss. Region I (30–120°C) indicates moisture release; regions II (120–250°C) and III (250–370°C) indicate two stages—thermal decomposition and evaporation; and region IV (370–550°C) indicates further thermal decomposition and combustion. In our study, we set the heating temperature at 100, 120 and 200°C to study in detail the phenomena in regions I and II of previous studies [28,29].

**Figure 3.**
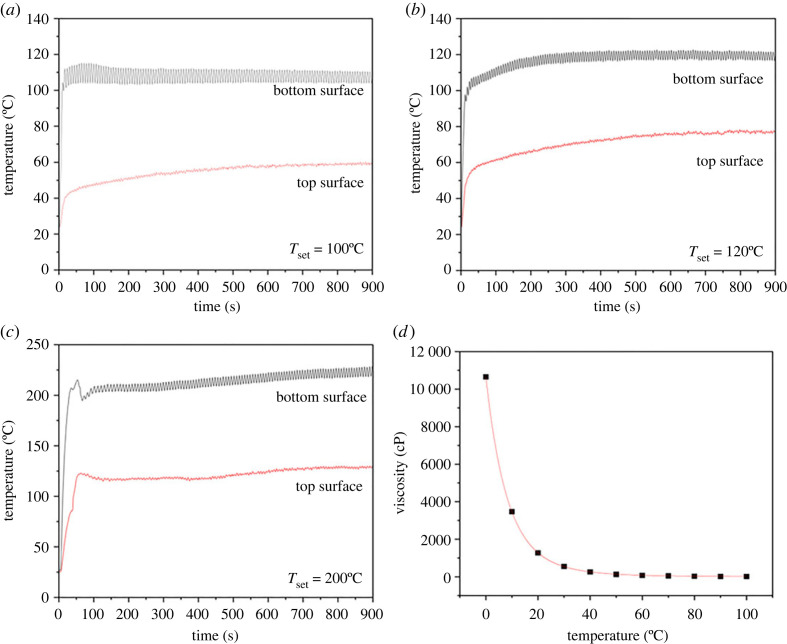
The temperature profile of the upper part of samples during heating at (*a*) 100°C, (*b*) 120°C and (*c*) 200°C; and (*d*) temperature dependence of glycerol viscosity (data fitted for glycerol 99.5% w/w from Segur & Oberstar [31]).

Figure 3*a–c* shows the temperature at the top and bottom surfaces of the tobacco substrates at each temperature set point with suction from the air pump. There was a significant difference between the bottom (direct contact with the heater) and the top (solid–air interface) surface temperatures of the tobacco substrates. The lower temperature at the top was due to forced convection at the solid–air interface activated by air pump suction. The evaporation of compounds such as water, glycerol and nicotine require energy, resulting in the cooling of the substrate [23]. Additionally, the Knudsen effect and phonon (heat carrier in solid) scattering at the solid–gas interphase within the pores of the sample's structure decreased the effective thermal conductivity of the sample [30], resulting in a lower temperature at the surface away from the heat source.

When glycerol was first dropped on the tobacco substrates, glycerol did not spontaneously wet and spread on the surface. The glycerol formed a sessile droplet with a diameter of approximately 3 mm (electronic supplementary material, figure S1). A contact angle at the glycerol, substrate and air interface was observed. The surface tension of glycerol was high, and the effect of viscous resistance was dominant at approximately 25°C (*µ* = ∼812 cP). At this stage, gravity counteracted viscous resistance because of the high mass per unit area of the glycerol.

As heating progresses, the contact angle disappears gradually. The time needed for the contact angle to disappear based on video observation is approximately 35 s for 100°C heating and approximately 25 s for 120 and 200°C heating. This time corresponds to tobacco surface temperature of 74.8, 85.2 and 95.5°C (figure 3*a–c*), and by assuming that the glycerol temperature equals surface temperature, the glycerol viscosity is approximately 36, approximately 23 and approximately 16 cP for 100, 120, and 200°C, respectively. The decrease in the viscosity of glycerol due to the increase in temperature, as fitted from the glycerol viscosity data at various temperatures provided by Segur & Oberstar [31], can be seen in figure 3*d*. Significant reduction in viscous flow resistance due to heating promotes glycerol spreading (movement of liquid in the *x*-axis direction) and penetration (movement of liquid in the *y*-axis direction), predominantly due to capillary forces.

Observation by the camera also revealed the time needed by liquid glycerol to spread to a maximum surface area. For heating temperatures of 100, 120 and 200°C, it took 240, 155 and 90 s, respectively, for glycerol to spread in-plane before a further change in surface area due to liquid spreading was undetected by the camera (electronic supplementary material, figure S2). The OCT system characterizes the change in the trans-planar (depth direction) of the substrate due to liquid flow. Because the scanning area of OCT is much smaller than the initial diameter of the glycerol on the surface of the sample, we expect that the variation in liquid distribution due to liquid spreading on the sample will not strongly influence the cross-sectional OCT scanning result. However, variations in scattered light from the sample may be observed by OCT when the point of observation is too far off the initial point of spreading.

**Figure 4.**
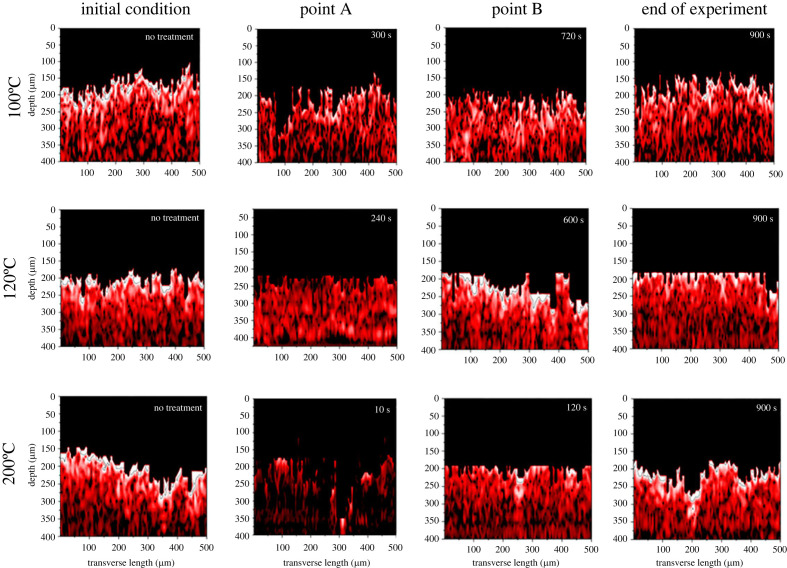
OCT tomograms of cross-sections of samples before, during and after heating at different temperatures.

Figure 4 shows four OCT tomograms of the porous tobacco substrates during the experiment. OCT tomograms help visualize the liquid flow in the media by capturing the cross-section image of the sample at each point in time. It is important to understand that the sample, a porous tobacco substrate, was produced by the papermaking process and is primarily composed of cellulose from tobacco stems and leaf scraps [32,33]. On average, the cellulose content of a large tobacco leaf stalk is 31–34%, while the tobacco leaf itself contains 16–17% cellulose [34]. Therefore, we assumed that cellulose was the major component of the sample. The refractive index (*n*) of cellulose is approximately 1.4613 (at *λ* = 1052 nm) [35].

At initial conditions, all tomograms show a bright (whitish) image on the surface of the sample due to air distribution inside the cellulose matrix of the tobacco substrates. Light scattering occurred when light travelled from the air (*n* = 1) to the tobacco substrates; the difference in the refractive indices of the two media caused intense scattering creating a bright tomogram.

The brightness of the tomogram reduces with time until approximately point A where the glycerol penetration is around its peak. At this point, the majority of the air inside the cellulose matrix had been displaced by the glycerol which had spread and penetrated due to capillary forces. The refractive index of glycerol is very close to the refractive index of cellulose in the near-infrared region—1.4631 (at *λ* = 1050 nm) [36]. The closeness of the refractive indices of glycerol and cellulose caused refractive index matching. As a result, the light was transmitted more effectively and deeper into the sample instead of being scattered on the surface. This resulted in lower backscattered light intensity [13,37,38]. Additionally, the glycerol absorbed some of the incident light, decreasing the intensity of the backscattered light further. The absorption coefficient of glycerol at 1470 nm wavelength is 10.113 cm^−1^ [39]. Makino *et al*. [14] used SD-OCT with two near infrared (NIR) wavelengths to see the evaporation process of water from the porous tobacco substrate under ambient conditions. They noted that the presence of water in the pores of the medium darkens the appearance of the tomographic image due to refractive index matching. Their finding supported the observation made in the present study.

It was also observed that the higher heating temperature in point A produced a darker tomogram, probably because the surface tension of glycerol dropped significantly, helping to wet tobacco substrates and displace air ‘completely’ in the cellulose matrix. Hence, the effect of refractive index matching became more pronounced and backscattering intensity decreased significantly [13].

After point A, evaporation became more dominant than liquid penetration. Capillary action distributed the liquid glycerol over a wide surface area, increasing the contact between the glycerol and air. This condition, coupled with the heating, allowed the majority of the glycerol to evaporate out of the sample far below its boiling point. Further heating evaporated more glycerol allowing air to re-enter the cellulose matrix. The presence of air in the cellulose matrix increased the backscattering intensity and terminated the effect of refractive index matching. This produced a brighter tomogram, as shown in point B and ‘end of experiment’ tomograms.

Liquid penetration and evaporation from the biomass substrate caused changes in the sample's morphology as seen, to an extent, in the appearance of the time-lapsed tomogram at each temperature. The ‘unnatural’ straight cuts on the surface of samples at 120°C (e.g. figure 4, first image from right) and 200°C (e.g. figure 4, second image from right) are attributed to the swelling of cellulose fibres when the glycerol penetrated the sample [7,13], and/or coiling of the samples when the glycerol evaporated. These morphological changes caused the sample to rise beyond the field of view of the OCT lens, resulting in ‘unnatural’ tomograms.

**Figure 5.**
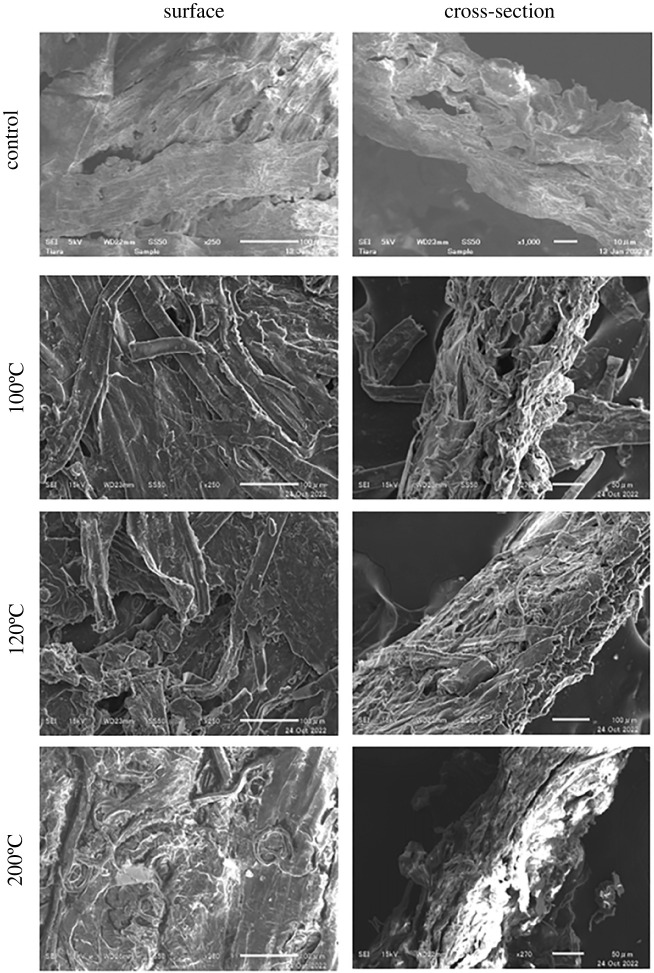
SEM images of the surface and cross-section of samples: before (control) and after heating at various temperatures.

SEM was also used to observe the change in the microstructure of the sample (figure 5). The SEM images suggest that the penetrating and evaporating glycerol shrunk some pores of tobacco substrate and ‘destroyed’ some of the cellulose fibre networks. This effect became stronger at higher temperatures. At the end of the heating experiment at 200°C, the tobacco substrates turned dark brown (electronic supplementary material, figure S2).

The movement of glycerol inside the tobacco substrates is quantified by calculating the attenuation coefficient using the scattered intensity data from the OCT system according to equation (2.2). The attenuation coefficient characterizes the strength of backscattering from the sample and is widely used to differentiate ‘healthy’ and ‘non-healthy’ tissues in clinical analysis [40–42]. A high attenuation coefficient represents high backscattering intensity while a low attenuation coefficient represents the contrary. In previous research [14], the movement of liquid water in porous tobacco substrate under ambient condition has been successfully quantified by the attenuation coefficient.

**Figure 6.**
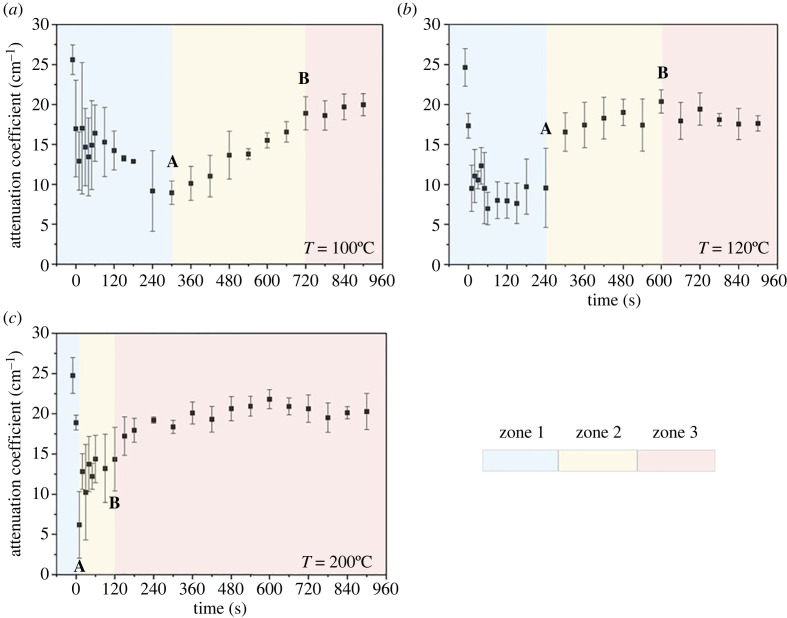
Temporal change in attenuation coefficient of tobacco substrates after dropping glycerol at: (*a*) 100°C, (*b*) 120°C and (*c*) 200°C. Values are mean ± s.d. of three independent experiments.

The attenuation coefficient plotted against elapsed time for each temperature is shown in figure 6*a–c*. Points A and B for each temperature were estimated using Duncan's test. The attenuation coefficient data (*n* = 3) at several time intervals of each heating temperature was used as the input. Duncan's test assigned each point in the dataset to one or more groups. The data that were significantly different from each other (*p* < 0.05) were assigned to different groups. Similar data shared the same group.

Point A is a time approximation where maximum penetration occurs before further heat transfer in an evaporation-dominant zone. Point A is the latest time in which the obtained attenuation coefficient is significantly different from the attenuation coefficient of the tobacco substrates at the initial condition (untreated sample). In Duncan's test result, critical point A belongs to the group farthest from the group where the attenuation coefficient of the untreated sample belonged.

Point B is a time approximation where liquid evaporation starts to slow down because most of the free liquid has evaporated. Point B is the earliest time in which the obtained attenuation coefficient becomes non-significantly different from the attenuation coefficient of the tobacco substrates at the initial condition (untreated sample). In Duncan's test result, critical point B belongs to the same group as the untreated sample.

It should be noted that points A and B are just approximation based on the significant difference relative to the initial condition that we propose in this first study. Variations in the timing of points A and B may occur. Additional study to address this issue is needed.

The two points divide the attenuation data into three different zones, namely zones 1, 2 and 3. Zone 1 (blue) is the penetration-dominant zone and begins immediately when heating starts after dropping the glycerol. Liquid penetration reaches a maximum at around point A, which occurs at approximately 300, approximately 240 and approximately 10 s for temperatures 100, 120 and 200°C, respectively. Heat transfer in this region is mainly sensible heat transfer and the energy gained by the sample increases the temperature of the sample and the glycerol within it. The temperature increase decreases the glycerol's viscous resistance making penetration due to capillary force dominant. Interestingly, there were little variations in the temperature difference between the top/upper and bottom surface (Δ*T*) of all samples during point A; average Δ*T* at point A = 53.1 ± 3.8°C.

Fabritius & Myllylä [13] tracked the sorption of glycerol in a paper medium using OCT at ambient temperature. By observing the OCT tomogram, they found that there is a 6.1 s delay before the capillary penetration begins. They noted that glycerol takes 10.3 s to penetrate from the front side to the back side of 420 µm paper. There is a deviation between the result of the observation in their study and the Washburn equation for the capillary flow of liquids in the porous medium due to the non-uniform pore size distribution of their medium. Additionally, the effect of substrate (multilayered biomass) temperature on the penetration of glycerol was investigated previously by Asanuma *et al*. [25] by using colloidal fluorescence particles and fluorescence microscopy. They found that for the same 1 min heating observation, glycerol droplet that is exposed to a higher temperature can penetrate deeper layers faster than the glycerol at a lower temperature. They also found that glycerol can spread more in the *x*-axis direction when the heating temperature is lower. Their observation validated the finding in the present study regarding the relationship between heating temperature and maximum penetration time. However, similar to the study by Fabritius & Myllylä, both studies identified the wetting front of the liquid but did not discuss the possibility that the pores behind the visible wetting front are not fully saturated. We expect that the gradual decrease of the attenuation coefficient before reaching to a maximum suggested a characterization of the state of intra-fibre pore filling behind the wetting front.

Cellulose fibre in tobacco substrates is typically made of micrometre-sized inter-fibre pores and nanometre-sized intra-fibre pores [3]. Inter-fibre pores are pores constructed from entangling individual cellulose fibres, while intra-fibre pores are the internal cavities in individual cellulose fibre. Liquid can be absorbed in both the inter-fibre and the intra-fibre pores [7], but the inter-fibre pores are more permeable because they are larger [3]. Hence, glycerol penetrates under capillary pressure into the inter-fibre pores and diffuses into the intra-fibre pores due to the concentration gradient. A detailed discussion on this phenomenon can be found in [7].

Energy accumulation in the sample after point A promotes glycerol evaporation due to latent heat transfer. Zones 2 (yellow; between A and B) and 3 (light red; after B) are evaporation-dominant zones. Zone 2 begins approximately after point A and ends at approximately point B; approximately 720, approximately 600 and approximately 120 s for temperatures 100, 120 and 200°C, respectively. Evaporation in zone 2 is considered the evaporation of free liquid in the sample matrix. Free liquid in this case is the glycerol which remained on the upper surface of the sample and in the inter-fibre pores.

After point B, there are slow and subtle changes in the attenuation coefficient. The drying rate is expected to decrease from point B because the liquid movement to the exposed surface is unable to replenish the liquid at the surface. At this point, most of the free liquid has evaporated and the ‘bound’ liquid combined with the ‘trapped’ liquid begins to evaporate. ‘Trapped’ liquid is defined as a liquid that is not ‘bound’ to the fibres but is difficult to transport to the exposed surface. Liquid trapped in nanoscale pores is considered a non-freezing bound liquid [43].

**Figure 7.**
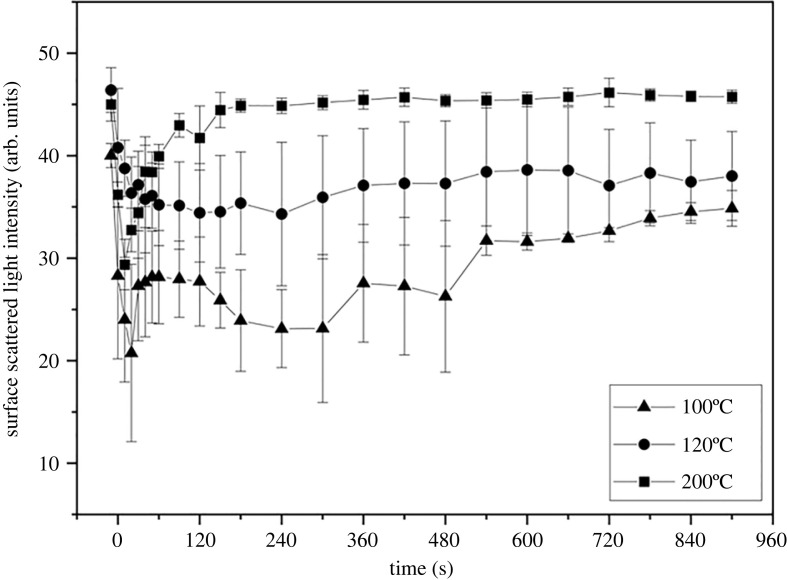
Scattered light intensity from the surface of the sample at various temperatures. Values are mean ± s.d. of three independent experiments.

To reduce the effect of structural change in our data interpretation, an analysis of light interaction with the surface of the sample is included. The upper sample surface is chosen because it is the first point of contact for the incident light before volumetric structural change further modifies the interaction between light and the sample. Additionally, the upper surface of the sample is assumed to be the main transport direction for the evaporating liquid, meaning if the surface is dry before the experiment is completed, it is reasonable to assume that there is no evaporation mass flux from below the surface and evaporation has terminated. To understand this phenomenon better, we plotted the backscattering intensity from the sample surface against the elapsed time at each temperature (figure 7).

In figure 7, the sample is considered dry only at 200°C after point B because the backscattered light intensity from the sample's surface after the point B was almost the same as that of the ‘dry’ (initial) sample, and there was no notable change until the end of the heating experiment. In addition, the attenuation coefficient is stable after point B even though it does not return to the ‘original’ attenuation coefficient value of the dry sample.

At 100 and 120°C, both the attenuation coefficient and backscattered light intensity from the surface value did not return to the sample's initial value. This indicates that the samples may not have been completely dried. The changes in attenuation coefficient after point B indicate slow evaporation of trapped liquid and bound liquid from the sample. The bound liquid is defined as the initial water [43] present in the sample, plus the glycerol bound to the hydrophilic surfaces of cellulose fibre by hydrogen bonds.

The phenomenon at the boundary between the sample's upper surface and the surrounding air is influenced by conduction from the heater to the sample and forced convection due to the air pump's suction flow. In some applications, such as food dehydration, convective drying results in low-porosity and high-density materials, which are undesired for some purposes [44]. For further studies, the dimensionless approach, for example, the Nusselt number (*Nu*), gives our research another advantage. For instance, *Nu* > 1 indicates that convective heat transfer influences the system more than conductive heat transfer. By increasing laminar flow in the chamber, convective heat transfer can be minimized.

The results of the present study could have implications for other porous/fibrous materials, such as papers and textiles, where capillary forces play a role in the penetration and spreading of liquids. The integration of a heating system and the use of attenuation coefficients calculated from OCT results to characterize liquid penetration and evaporation is expected to shed light on a better understanding of dynamic liquid flow in a medium with a non-uniform pore size whose behaviour deviates from the Washburn equation.

In the future, it is expected that a heating system such as the one proposed in the present study, can be integrated with functional extensions of OCT such as phase-sensitive/polarization-sensitive (PS) and Doppler OCT for a more thorough understanding on the dynamics of temperature-dependent liquid flow phenomena in porous materials that are hundreds of micrometres thick. PS OCT provides not just imaging based on tissue reflectivity but also enables the mapping of sample polarization properties with depth resolution [45]. By analysing the changes in polarization as the light passes through the porous material, PS OCT can distinguish between areas with and without the liquid. Doppler OCT on the other hand, allows the quantification of velocity as well as the direction of flow within a tissue by applying Doppler principle, in addition to structural imaging [46]. By measuring the Doppler frequency shift, one can try to quantify the temperature-dependent velocity of liquid imbibition in porous materials. Additionally, based on more recent developments, the speckle patterns (i.e. random patterns of bright and dark spots that appear when coherent light interacts with a rough or scattering surface) from OCT [47] can be further analysed to characterize liquid-filled porous materials.

## 4. Conclusion

OCT system coupled with a heating chamber connected to an air pump suction line has allowed an unprecedented, non-destructive and real-time observation of the penetration and evaporation of viscous liquid in porous biomass during heating. The movement of liquid in a porous tobacco substrate can be observed through the OCT tomogram at several time lapses and can be quantified by the attenuation coefficient of the sample at a given temperature and time. By testing the time-lapse attenuation coefficient data using a statistical procedure (Duncan's multiple range test), we were able to approximate the transitional points in penetration and evaporation at each temperature. The proposed analytical system is an attractive alternative for tracking liquid transport in porous biomass when contact heating is applied, and is also useful for understanding inward and outward mass transfer.

## Data Availability

The ‘downloadable’ dataset is available at Dryad: https://doi.org/10.5061/dryad.kprr4xh8j [48]. The data are provided in electronic supplementary material [49].
